# P24 A case of limited cutaneous systemic sclerosis evolving into an overlap syndrome with systemic lupus erythematosus and pyoderma gangrenosum

**DOI:** 10.1093/rap/rkac067.024

**Published:** 2022-09-28

**Authors:** Gagandeep Sukhija, Yik Long Man, Pamela Lutalo, Louise Pollard, Ghada Yanni

**Affiliations:** University Hospital Lewisham, Lewisham and Greenwich NHS trust, London, United Kingdom; University Hospital Lewisham, Lewisham and Greenwich NHS trust, London, United Kingdom; University Hospital Lewisham, Lewisham and Greenwich NHS trust, London, United Kingdom; University Hospital Lewisham, Lewisham and Greenwich NHS trust, London, United Kingdom; University Hospital Lewisham, Lewisham and Greenwich NHS trust, London, United Kingdom

## Abstract

**Introduction/Background:**

Systemic sclerosis is an autoimmune condition characterised by fibrosis of the skin and internal organs. A considerable number of patients with systemic sclerosis may present with symptoms of other connective tissue disorders over time. The onset of overlap syndromes affects management and prognosis. We present a case of a 46-year-old man with limited cutaneous systemic sclerosis who presented with significant proteinuria secondary to lupus nephritis, and non-healing leg ulcers secondary to pyoderma gangrenosum, a condition that is rarely associated with SLE or systemic sclerosis. To our knowledge there are no published reports of SSc-SLE-Pyoderma Gangrenosum Overlap.

**Description/Method:**

In 2004, a 28-year-old Black man presented to the rheumatology clinic with a history of Raynaud’s phenomenon, digital ulcers, sclerodactyly and widespread telangiectasiae. He was ANA (nucleolar pattern), anti-Ro and anti-Jo1 positive, however he did not have clinical features of Sjogren’s syndrome or anti-synthetase syndrome. He was diagnosed with limited cutaneous systemic sclerosis and managed with Nifedipine and Sildenafil. Over the next decade of rheumatology clinic monitoring he did not develop pulmonary hypertension or organ involvement.

In 2018, he was admitted to hospital with a pulmonary embolus and had a negative thrombophilia screen and antiphospholipid syndrome screen. Immunology revealed new autoantibodies: anti-Smith, Anti-U1RNP and anti-dsDNA.

In 2021, aged 46-years, he was admitted to hospital with a large, painful punched-out ulcer on his left medial malleolus that was rapidly expanding. In addition, he had noticed significant weight loss and breathlessness on minimal exertion. He was hypoalbuminemic (albumin = 30g/l [35-52] ) with proteinuria (urine protein:creatinine ratio 150mg/mmol), however his creatinine and eGFR were normal. Immunology was unchanged. He had hypocomplementenemia. He was ANCA and Cryoglobulin negative. CT chest-abdomen-pelvis showed small pleural effusions but no malignancy or infection. Echocardiogram showed a small pericardial effusion with normal pulmonary artery pressures. Vascular doppler scans showed normal arterial circulation in his lower limbs. Microbiology reported a significant growth of Pseudomonas Aeruginosa from the leg ulcer swab and the skin biopsy showed neutrophilic infiltrates in the dermis, consistent with pyoderma gangrenosum. Renal biopsy was consistent with lupus nephritis class III and V.

In-patient management included a course of antibiotics for the Pseudomonas Aeruginosa infection followed by oral Prednisolone and Mycophenolate Mofetil for lupus nephritis. The proteinuria has resolved and the left leg ulcer has reduced in size however he has developed new ulcers on the right leg which are being treated at present.

**Discussion/Results:**

Overlap connective tissue disorders are well recognised in rheumatology. This patient initially presented with features of limited cutaneous systemic sclerosis (Raynaud’s phenomenon, digital ulcers and sclerodactyly). Limited cutaneous systemic sclerosis is typically associated with anti-centromere antibodies, but it can also be associated with nucleolar staining pattern of ANA or anti-Ro antibodies, which was seen in this patient. On presentation with a pulmonary embolus in 2018, his antibody profile showed anti-dsDNA, anti-Smith, anti-U1RNP, low complements although he had no clinical features suggestive of SLE. When he later presented with a leg ulcer, he had serositis and a raised protein-creatinine ratio, more consistent with SLE. He was normotensive and had a normal creatinine, which would go against a scleroderma renal crisis, and indeed the renal biopsy confirmed lupus nephritis. The incidence of overlap between SLE-SSc has been found to be 8.4% to 14.4% in some studies. Previous studies have shown that patients with SLE-SSc overlap have less frequent skin manifestations whereas our patient had florid telangiectasiae.

Pyoderma gangrenosum is an uncommon ulcerative skin disease which has a well-recognised association with inflammatory bowel disease but it can also be seen with autoimmune rheumatic diseases. It is rarely seen with SLE and limited systemic sclerosis and has not been reported with an overlap syndrome. It is important to exclude chronic vascular ulcers, pyodermatitis and non-infectious aetiologies like vasculitis. Vasculitis was an important differential diagnosis in this case, as the leg ulcers emerged at the same time as lupus nephritis. Vasculitis was excluded as a diagnosis based on the biopsy and immunology reports. This case demonstrates an uncommon association between pyoderma gangrenosum with an SSc-SLE overlap syndrome. Pyoderma gangrenosum responds well to systemic steroids or immunosuppressants, therefore it is unusual that the leg ulcers are progressing. He has been referred for further investigations.

**Key learning points/Conclusion:**

Limited cutaneous systemic sclerosis is characterised by calcinosis, Raynaud’s phenomenon, oesophageal dysmotility, sclerodactyly and telangiectasiae (previously known as CREST). It’s association with anti-centromere antibodies is well recognized, although studies have shown that it is only positive in 20-30% of patients with limited SSc. It is important to remember that limited SSc may be only ANA positive, but can also be associated with other auto-antibodies, including anti-Ro, as highlighted by this case.

An overlap syndrome is defined as a disease complex when the classification criteria for two different connective tissue diseases are satisfied. The diseases may present simultaneously or at different time points in the patient journey, as is the case with this patient who was diagnosed with SLE almost 2 decades after his initial diagnosis with limited cutaneous systemic sclerosis. Among scleroderma overlap syndromes SSc-SLE is the second most common after SSc-polymyositis. This case highlights the importance of repeating the serology when the clinical phenotype changes. It is beneficial to recognise overlap syndromes as they require alternative treatment strategies and early intervention improves prognosis.

SSc-SLE overlap syndrome demographic data shows predominance in female patients; patients of South and East Asian ethnicity and patients with disease onset at a young age.

SSc-SLE clinical manifestations include musculoskeletal disease (62.5%) and limited cutaneous systems sclerosis (32.2%) with lung and cardiac involvement being reported in young patients.

Pyoderma gangrenosum is usually diagnosed after excluding infectious and noninfectious causes of cutaneous ulcers. Diagnosis requires a skin biopsy showing inflammatory neutrophilic infiltrates in the dermis or a response to steroid therapy. It is important to recognise this condition early so that clinically appropriate treatment is initiated and prognosis improves.

